# IL-27 attenuates airway inflammation in a mouse asthma model via the STAT1 and GADD45γ/p38 MAPK pathways

**DOI:** 10.1186/s12967-016-1039-x

**Published:** 2016-09-29

**Authors:** Xiaoqiong Su, Jue Pan, Fengxi Bai, Honglei Yuan, Nian Dong, Dandan Li, Xiangdong Wang, Zhihong Chen

**Affiliations:** 1 Respiratory Division of Zhongshan Hospital, Shanghai Institute of Respiratory Disease, Fudan University, No.180 Fenglin Road, Shanghai, China; 2 Research Center of Zhongshan Hospital, Fudan University, No 180 Fenglin Road, Shanghai, 200032 China; 3 Department of Pulmonary Medicine, The First Affiliated Hospital, Wenzhou Medical University, Wenzhou, China

**Keywords:** IL-27, Th2 development, STAT1, GADD45γ, p38 MAPK

## Abstract

**Background:**

Asthma is prone to Th2-mediated chronic airway inflammation. Interleukin-27 (IL-27) is a member of the IL-12 family that promotes the differentiation of Th1 cells and inhibits Th2 cells. We use human/mouse CD4^+^ T cells to see whether IL-27 could inhibit IL-4 production in vitro and then observe whether IL-27 administration could alleviate allergic airway inflammation in vivo by mice asthma model.

**Methods:**

We isolated and cultured CD4^+^ T cells from healthy humans and mice to test whether IL-27 could inhibit IL-4 production under different conditions. In vivo study, the effect of IL-27 was examined using two types of intra-nasal (i.n.) administration: low-dose-multiple-times prevention or high-dose-limited-times treatment in murine asthma models. The expression levels of signal transducer and activator of transcription-1 (STAT1) and growth arrest and DNA damage 45-γ (GADD45γ)/p38 mitogen activated protein kinase (p38 MAPK) in lung tissues were measured using qPCR and Western blotting.

**Results:**

In vitro, although IL-27 could inhibit naïve CD4^+^ T cell differentiate into Th2 cells, but it could not redifferentiate already committed Th2 cells. In vivo, preventative administration of IL-27 attenuated allergic inflammation and airway hyperreactivity, whereas treatment group had no significant effect. In the asthma group, the phosphorylation of STAT1 was impaired, while GADD45γ and p38 MAPK exhibited no obvious changes. Preventative administration of IL-27 could either reverse the impairment of STAT1 or strengthen the expression of GADD45γ and p38 MAPK, whereas treatment group had no significant effect.

**Conclusions:**

Preventative administration of IL-27 improved the pathological changes in mouse asthma models via both the STAT1 and GADD45γ/p38 MAPK pathways while therapeutic administration of IL-27 had no significant effect, which may be due to the presence of already differentiated Th2 cells in asthmatic airways that resist IL-27 inhibition.

**Electronic supplementary material:**

The online version of this article (doi:10.1186/s12967-016-1039-x) contains supplementary material, which is available to authorized users.

## Background

According to the “Hygiene Hypothesis” that has been proposed in the early years, the imbalance of Th1 and Th2 responses in T lymphocytes and the tendency toward a Th2 response is the primary driver of airway inflammation pathogenesis in asthma [1]. Although established Th cells exhibit some plasticity, especially under specific circumstances such as infections, Th cells that have already committed to one Th cell fate tend to down-regulate their potential to differentiate into other Th cells. For example, significant experimental evidence supports the theory that committed Th1 cells minimize their potential to transcribe the IL-4 gene [2]. For example, the T-box transcription factor (T-bet), which is critical in Th1 differentiation, possesses the ability to suppress IL-4 gene transcription. In addition to T-bet, it has been reported that signal transducer and activator of transcription (STAT)4, or interferon regulatory factor-1 and -2 (mice deficient in Th1-promoting factors) demonstrate the propensity to mount a Th2-type immune response against pathogens [3, 4]. These results clearly demonstrate that Th1-promoting factors are critical in suppressing Th2 cell differentiation.

IL-27, a member of IL-12 family, is a heterodimeric cytokine composed of EBV-induced gene 3 (EBi3) and p28, and it is primarily produced by activated macrophages and dendritic cells [5]. Previous data demonstrated L-27R–deficient mice displayed increased Th2 production when infected with the parasites Leishmania major or *Trichuris muris* [6]. Transgenic expression of IL-27 suppressed TH2 responses induced by *Strongyloides venezuelensis* [7]. STAT1 is required for immunity against bacteria and viruses in mice [8]. Human subjects deficient in STAT1 die of bacterial and viral infections at an early age [9, 10]. We demonstrated that IL-27, a key cytokine in generating immunity against bacterial and viral infection, also depends on STAT1 to suppress TH2 cell differentiation. GADD45γ is a member of a group of genes whose transcript levels are increased following treatment with DNA-damaging agents. GADD45γ is induced during T cell activation and that the level of expression is higher in Th1 cells than in Th2 cells [11].

Of note, recent research has indicated that IL-27 can’t inhibit already differentiated Th17 cells [12], and our previous study indicated CD4^+^ T cells from asthmatic patients resisted IL-27-mediated suppression of IL-4 production [13, 14].

The present study aimed to determine whether IL-27 can inhibit differentiated Th2 cells in vitro. Furthermore, we investigated the effects of preventive and therapeutic IL-27 administration on the allergic airway inflammation in ovalbumin (OVA)-induced asthma mouse models and studied whether the effect is related to the STAT1 and GADD45γ/p38 MAPK pathways.

## Methods

### CD4^+^ T cell culture and IL-4 protein measurement

For the human CD4^+^ T cells, peripheral blood mononuclear cells (PBMCs) were isolated using Ficoll-Hypaque (Histopaque-1077; Sigma-Aldrich, St Louis, MO, USA) gradient centrifugation from the 15 mL of peripheral blood collected from each healthy volunteer. The CD4^+^ T cells were isolated from the PBMCs by means of magnetic bead separation (magnetic cell sorting) with a human CD4^+^ T Cell Isolation Kit (Miltenyi Biotec). We routinely obtained greater than 90 % CD4^+^ T cell purity. The neutralizing conditions contained 0.5 mg/mL of anti-human CD3 antibody, 1 mg/mL of anti-human CD28 antibody, 50 U/mL of rhIL-2, 10 mg/mL of anti-human IFN-γ antibody, and 10 mg/mL of anti-human IL-4 antibody. The Th2-inducing conditions contained 0.5 mg/mL of anti-human CD3 antibody, 1 mg/mL of anti-human CD28 antibody, 50 U/mL of rhIL-2, 10 mg/mL of anti-human IFN-γ antibody, and 15 ng/mL of rhIL-4. After a 6-day culture period, the resultant cells were washed to remove cytokines that were added to prime the CD4^+^ T cells, and the cells were then stimulated overnight with phorbol 12-myristate 13-acetate (50 ng/mL) and ionomycin (1 mmol/L) to induce cytokine protein synthesis. The supernatants were collected, and the IL-4 protein concentrations were measured using ELISA. We obtained mononuclear cells from the mouse spleens by extracting the red cells using a red blood cell lysis buffer (E00016-1647, eBioscience, San Diego, CA, USA) and continuing with the steps used for isolating human PBMCs.

### Mice

Six-week-old female C57/BL6 mice (Silaike experimental animal limited liability company, Shanghai, China) were housed in pathogen-free conditions. All of the animal experiments were approved by the Animal Care and Use Committee at the Zhongshan Hospital Affiliate of Fudan University, Shanghai, China. The protocol (No: B2014-108) was previously approved by the Institutional Review Board at Fudan University.

### Reagents

OVA powder was purchased from Sigma-Aldrich (Grade V; St Louis, MO, USA). Adjuvant aluminum hydroxide (Al(OH)3(Alum)) was obtained from Thermo Fisher Scientific (Waltham, MA, USA). Recombinant human IL-27 antibody (rhIL-27), recombinant mouse IL27 antibody (rmIL-27), recombinant human IL-4 antibody (rhIL-4), recombinant mouse IL-4 antibody (rmIL-4), recombinant human IL-2 antibody (rhIL-2), recombinant mouse IL-2 antibody, anti-human INF-γ antibody (rmIL-2), anti-mouse INF-γ antibody, anti-human IL-4 antibody and anti-mouse IL-4 antibody were obtained from R&D Systems (Minneapolis, MN, USA). Anti-human CD3 antibody, anti-mouse CD3 antibody, anti-human CD28 antibody and anti-mouse CD28 antibody were obtained from eBioscience (San Diego, CA, USA). The following antibodies were also used: anti-STAT1 antibody (SC592; Santa Cruz Biotechnology, Santa Cruz, CA, USA); anti-Py-STAT1 antibody (catalog no. 9171; Cell Signaling, Boston, MA, USA), anti-GADD45γ antibody (ab196774,Abcam,Cambridge,UK), anti-p38 MAPK antibody (CST9212, Cell Signaling, Boston, MA, USA), and anti-phospho-p38 MAPK antibody (CST9215, Cell Signaling, Boston, MA, USA). The concentrations of IL-4, IL-5, and IL-13 were determined using ELISA kits (eBioscience).

### Ovalbumin-induced allergic asthma model and prevention and treatment regimens

Specific protocols for each mouse model are shown in Figs. 2a, 3a, 4a and 5a. Briefly, for the asthma model, 6-week-old female C57/BL6 mice were sensitized with an intraperitoneal injection (i.p.) of 100 µL of phosphate-buffered saline (PBS) (Invitrogen, Paisley, Scotland or USA) solution containing 100 µg OVA with 2 mg of Alum on days 0 and 7. From days 14 to 18, the mice were challenged on 5 consecutive days via an intra-nasal (i.n.) administration of 100 µg of OVA in 50 µL of PBS under light isoflurane anesthesia [15]. In the limited high-dose treatment group, the mice were treated i.n. with IL-27 (1 µg/mouse) once a day following the last 3 days of OVA -challenge. In the multiple low-dose prevention group, the mice were treated i.n. with IL-27 at two concentrations (25 and 50 ng/mouse) twice a day for 14 days before the second OVA sensitization. There were a minimum of eight mice in each group.

### Analysis of bronchoalveolar lavage (BAL) fluid in asthma mice model

The mice were sacrificed within 24 h after the last OVA challenge, and a BAL was immediately performed using 3 × 1 mL of 0.05 mM PBS–EDTA (Calbiochem, Darmstadt, Germany) as previously described [16, 17]. The cells were recovered through gentle manual aspiration. After centrifugation (1200 rpm for 10 min, at 4 °C), the supernatant was collected and frozen at −80 °C for IL-5 and IL-13 assessment by ELISA. The differential cell counts were determined from Wright–Giemsa-stained cytospins.

### Asthma mice model pulmonary histologic staining and scoring

After the BAL, the right main bronchus was clamped, and the left lung was excised. The left lung was infused with 4 % paraformaldehyde and embedded in paraffin. Five-micrometer paraffin sections were stained with hematoxylin–eosin (H&E) or periodic acid–Schiff (PAS). The right lung was collected and frozen at −80 °C for protein assessment using Western blot. The extent of peribronchial and inflammatory-cell infiltration around the bronchi in the lung tissue was estimated with a score calculated by quantification of the peribronchial and perivascular inflammatory cells, such as eosinophils, lymphocytes, and macrophages, as previously described [18]. Briefly, a value of 0 was attributed when no inflammatory cells were detectable around the bronchi. A value of 1 was attributed when there were occasionally inflammatory cells, a value of 2 was used when most bronchi were surrounded by a thin layer (one to five cells) of inflammatory cells, and a value of 3 was used when most bronchi were surrounded by a thick layer (more than five cells) of inflammatory cells. The same method was used for the perivascular inflammatory cells. Five to seven randomly selected tissue sections were scored per mouse, and thus the peribronchial and perivascular inflammation scores are expressed as a mean value per animal and can be compared between the groups in this study. These slides were evaluated and scored by two independently pathologists.

### Measurement of airway hyperreactivity (AHR) in asthma mice model

The mice were anesthetized for measurement of their pulmonary mechanics (Buxco Electronics, Wilmington, NC) 24 h after the last OVA challenge, as previously described [19]. Briefly, the mice were anesthetized with 50 mg/kg of pentobarbital and were wired to measure their pulmonary mechanics (BUXCO Electronics). The mice were tracheostomized, intubated, and mechanically ventilated at a tidal volume of 0.2 mL and a frequency of 150 breaths/min as previously described [20]. The lung resistance (RL) and Dynamic lung compliance (Cydn) were measured in response to increasing doses (0–20 mg/mL) of aerosolized acetyl-β-methylcholine chloride (methacholine) (Sigma-Aldrich).

### Measurement of gene expression

The total RNA was isolated using a guanidinium isothiocyanate/chloroform-based technique (TRIZOL, Invitrogen, USA) and was measured at an OD of 260 nm. The RNA was subsequently reverse transcribed into cDNA with the SuperScript First-strand Synthesis System (Invitrogen, USA). Quantitative RT-PCR was conducted using an ABI 7000 PCR instrument (Eppendorf, Hamburg, Germany) with two-stage program parameters as follows: 1 min at 95 °C, followed by 40 cycles of 5 s at 95 °C and 30 s at 60 °C. The sequences of the primer sets used for this analysis were as follows: STAT1, 5′-TATTCCAGACCAAAGGAAGCAC-3′ (forward [F]) and 5′-GAAGGGTGGACTTCAGACACAG-3′ (reverse [R]); GADD45-γ, 5′-TCTACGAGTCCGCCAAAGTC-3′ ([F]) and 5′-GCACTTGCCACTGGTGTAGA-3′ ([R]); p38 MAPK, 5′-CTATGGCTCGGTGTGTGCT-3′ ([F]) and 5′-GACGCAACTCTCGGTAGGTC-3′ ([R]); and mouse glyceraldehyde-3-phosphate dehydrogenase (GAPDH) (Sangon, Shanghai, China). The specificity of the produced amplification product was confirmed using dissociation reaction plots. Each sample was tested in triplicate with quantitative RT-PCR, and there were at least three wells for each group.

### Statistical analysis

All of the error bars in this report represent SDs. For the ELISA or Western blot analyses, the mean ± SD was derived from triplicate measurements of one experiment. The pooled data are represented in the figure legends. Differences between the data sets were analyzed using a one-way ANOVA with a Bonferroni post-test to determine significance (Prism 5; GraphPad Software, San Diego, CA, USA). A P value < 0.05 was considered statistically significant.

## Results

### Differentiated Th2 cells resist IL-27-mediated inhibition of IL-4 production

**Fig. 1:**
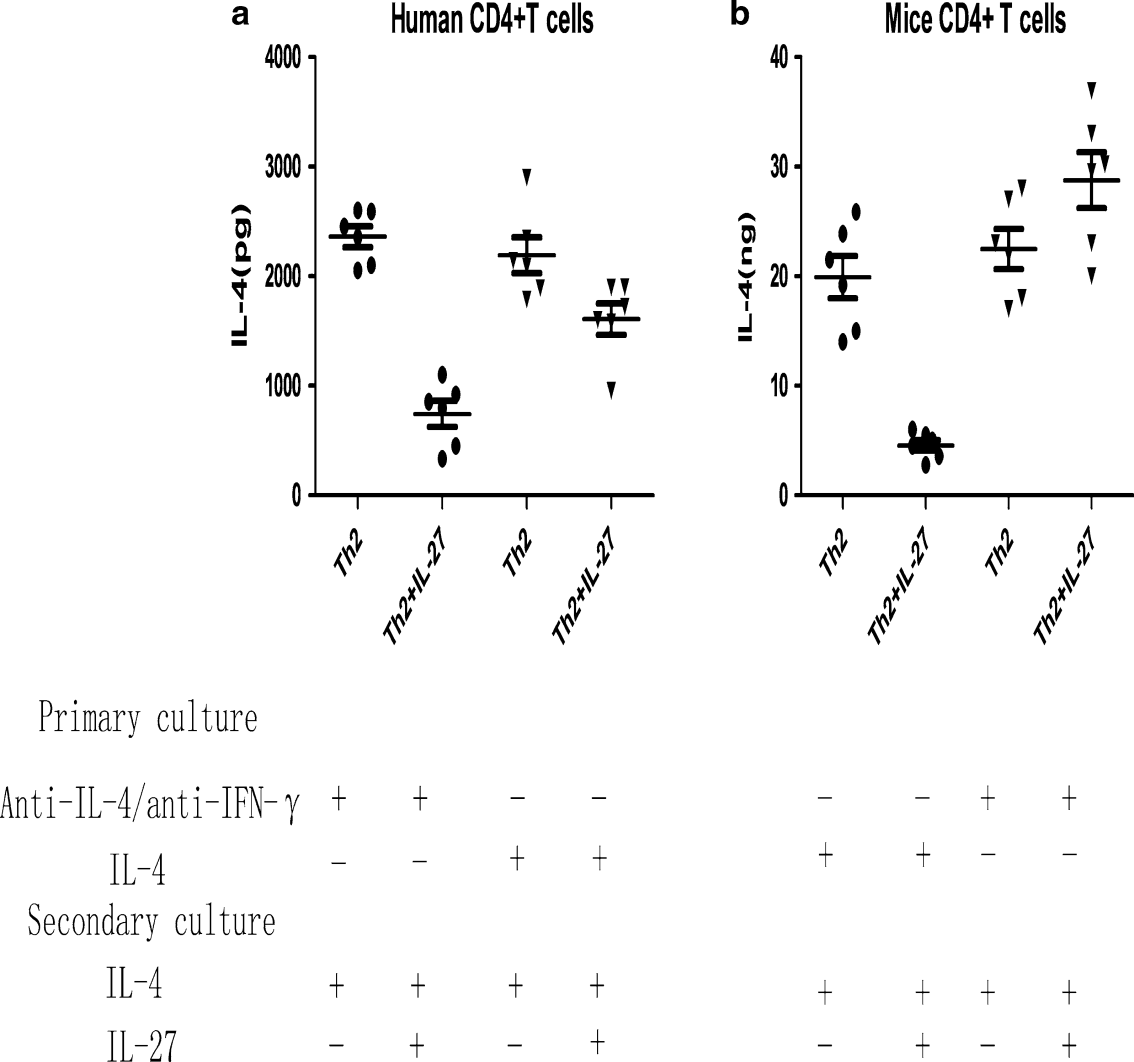
Repeated exposure to TH2-inducing conditions induces IL-27 resistance. **a** ELISA analysis of IL-4 produced by healthy human CD4^+^ T cells subjected to two rounds of priming. **b** ELISA analysis of IL-4 produced by mouse CD4^+^ T cells primed as indicated. Error bars and statistical analyses are described in the “Methods” section. *P < 0.05, **P < 0.01, ***P < 0.001. The data represent more than three independent experiments with similar results

In our previous study, we found that CD4^+^ T cells from asthmatic patients resisted the suppression of IL-4 production mediated by IL-27 [13]. A possible cause for this finding is that CD4^+^ T cells from healthy subjects are mostly made up of naive CD4^+^ T cells, whereas CD4^+^ T cells from allergic asthmatic patients are predominantly Th2 cells resulting from repeated exposure to allergen stimulation. It is possible that after repeated exposure to allergen stimulation, CD4^+^ T cells in the peripheral blood of allergic asthmatic patients develop resistance to IL-27-mediated suppression. To examine this hypothesis, we repeatedly primed CD4^+^ T cells from healthy subjects under Th2-inducing conditions. As a control, CD4^+^ T cells from healthy subjects were first primed under neutralizing culture conditions (containing anti-IL-4 and anti-IFN-γ antibodies) and were then primed under Th2-inducing conditions. We found that repeated Th2 priming enabled CD4^+^ T cells from healthy subjects to resist IL-27–mediated inhibition (Fig. 1a). We verified this finding with a murine Th2 differentiation system. We showed that murine IL-27 inhibited the differentiation of naive CD4^+^ T cells into Th2 cells but did not inhibit IL-4 production in differentiated Th2 cells (Fig. 1b). Thus, we concluded that differentiated Th2 cells resist IL-27-mediated inhibition of IL-4 production.

### Therapeutic intranasal administration of IL-27 had no significant improvement of airway inflammation in an OVA-induced mouse model

**Fig. 2:**
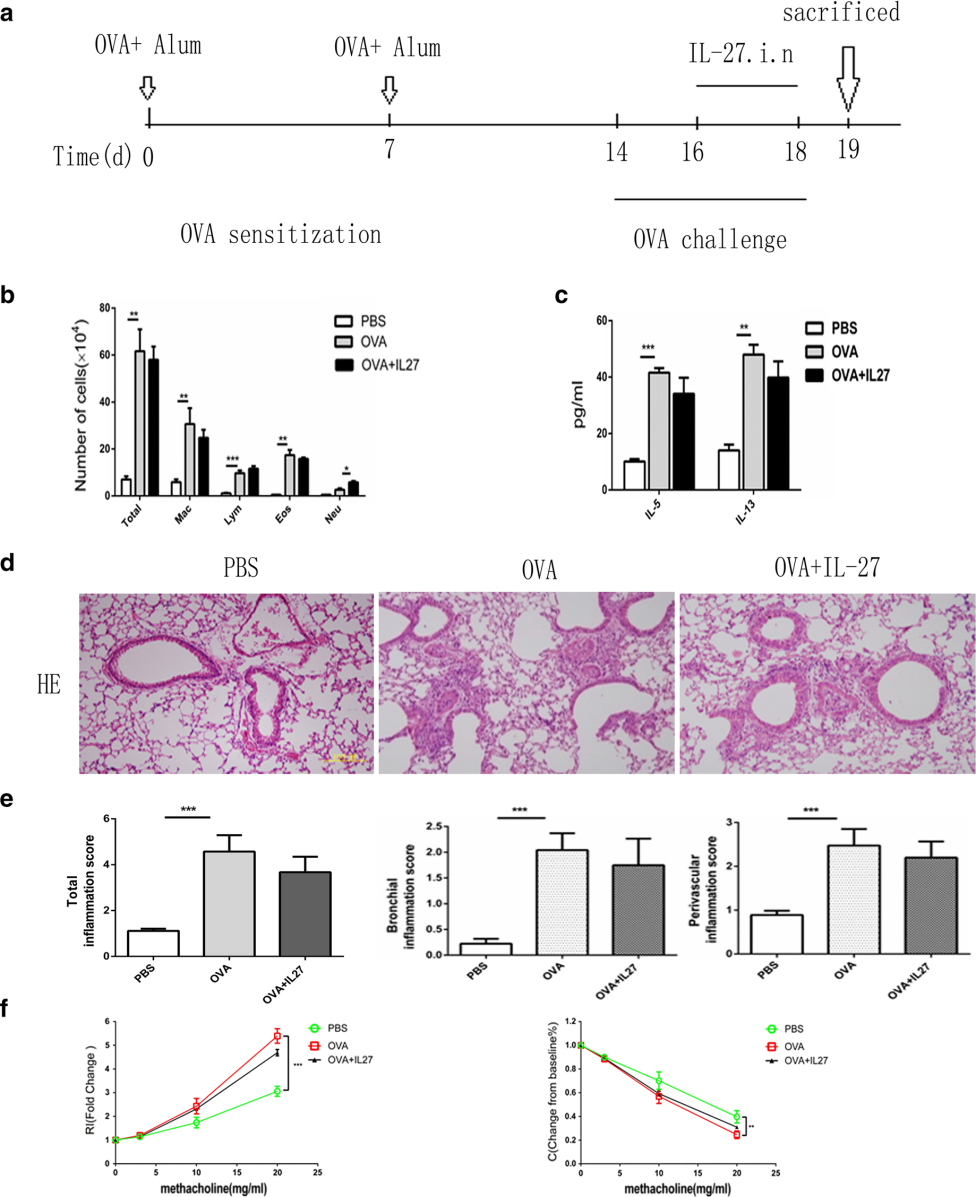
Therapeutic intranasal administration of IL-27 does not result in significant improvement of airway inflammation in an OVA-induced mouse model. **a** Protocol of OVA-induced allergic asthma and IL-27 administration. **b** The total cell number and the differential cell counts in bronchoalveolar lavage fluid (BALF). The total cell number and the differential cell counts had no significant difference in IL-27 treated group. **c** The concentrations of Th2 cytokines in BALF were measured by ELISA. IL-5 and IL-13 level was not markedly changed in IL-27 treated group. **d** Representative photomicrographs of lung sections stained with H&E were examined. There was no improvement of inflammation around airway and vessels in IL-27 treated group. **e** The inflammation scores of peribronchial and perivascular inflammation cells didn’t decreased in IL-27 treated group. **f** AHR was measured by invasive measurement of lung resistance in response to increasing concentrations of methacholine. IL-27 treatment had no improvement in lung resistance. The *columns* and *error bars* represent the mean and SEM (n = 6 per group). *P < 0.05, **P < 0.01, ***P < 0.001. Similar results were obtained in at least six independent experiments

Having demonstrated that differentiated Th2 cells resist IL-27-mediated inhibition in vitro, we wanted to further investigate whether the inhibition effect works in vivo in a mouse asthma model. We first administered IL-27 to OVA-immunized mice in the last 3 consecutive days of treatment (Fig. 2a). IL-27 treatment did not significantly attenuate airway inflammation and did not significantly decrease the total cell numbers and differential cell counts in BALF (Fig. 2b) or the concentration of IL-5 and IL-13 in BALF (Fig. 2c). Furthermore, there was no obvious reduction in the peribronchial and perivascular inflammatory infiltration in H&E-stained lung sections (Fig. 2d); the inflammatory infiltration level was further quantitated via inflammation scores (Fig. 2e). Moreover, for the methacholine-induced AHR, a hallmark of asthma, the IL-27 treatment also offered no significant protection (Fig. 2f). Together, these results demonstrate that differentiated Th2 cells in the complex asthma environment also resist IL-27-mediated inhibition in vivo. Further, limited high-dose administration of exogenous IL-27 in the challenge stage of asthma did not effectively suppress Th2-mediated allergic asthma.

### Preventative intranasal administration of IL-27 in an OVA-induced mouse model resulted in significant improvement

**Fig. 3:**
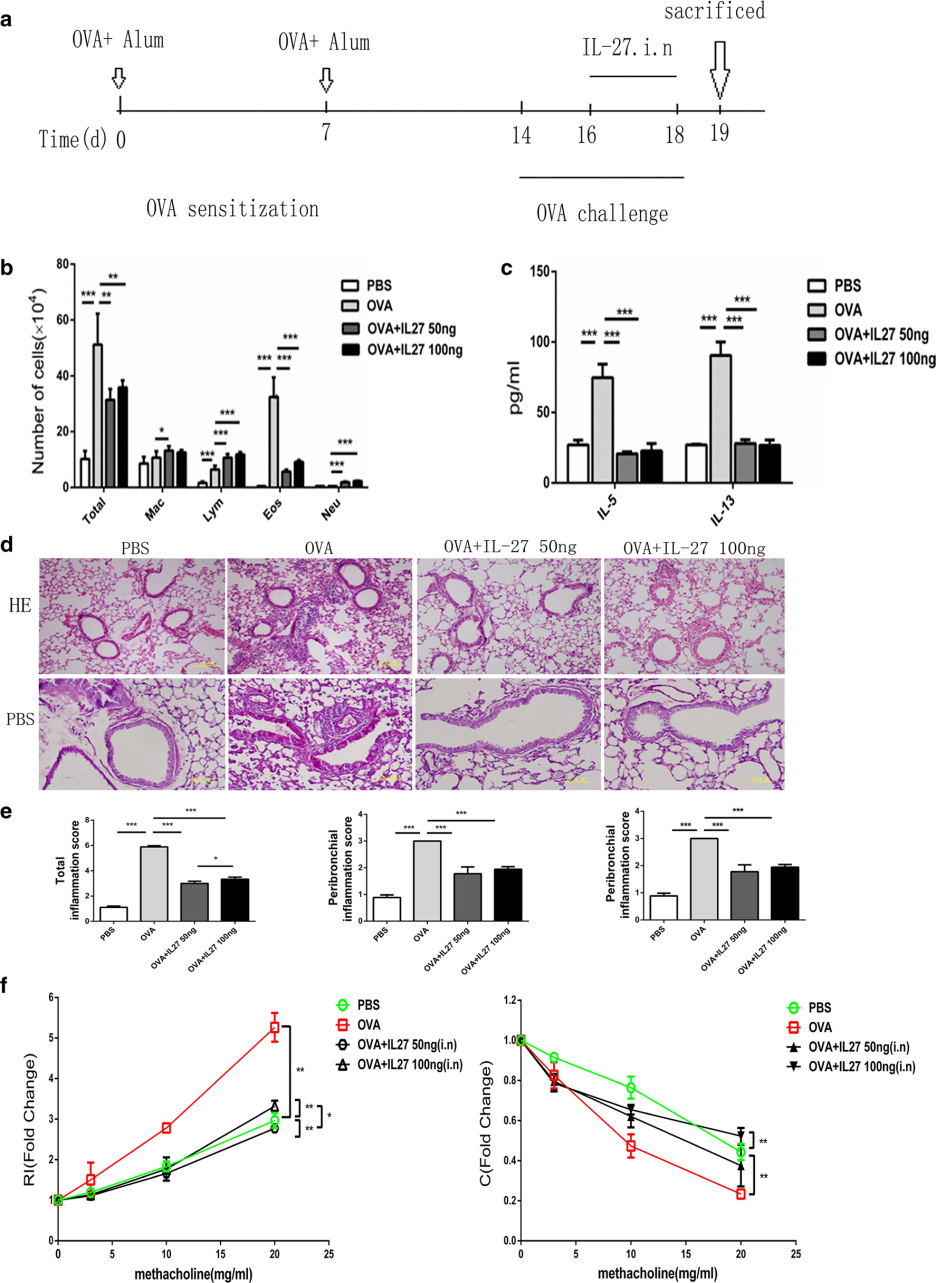
Preventative intranasal administration of IL-27 results in obvious improvement in an OVA-induced mouse model. **a** Protocol of OVA-induced allergic asthma and IL-27 administration. **b** The total cell number and the differential cell counts in bronchoalveolar lavage fluid (BALF). The total cell number and eosinophil number were decreased in IL-27 treated group. **c** The concentrations of Th2 cytokines in BALF were measured by ELISA. IL-5 and IL-13 level was lower in IL-27 treated group than OVA group. **d** Representative photomicrographs of lung sections stained with H&E or PAS were examined. IL-27 preventative treatment alleviated lung inflammation. **e** The inflammation scores of peribronchial and perivascular inflammation cells were markedly lower in IL-27 preventatively treated group. **f** AHR was measured by invasive measurement of lung resistance in response to increasing concentrations of methacholine. IL-27 preventatively treated group was seen the improvement of lung resistance. The *columns* and *error bars* represent the mean and SEM (n = 6 per group). *P < 0.05, **P < 0.01, ***P < 0.001. Similar results were obtained in at least six independent experiments

It is possible that the differentiated Th2 cells mediated the lack of improvement of airway inflammation in the asthma mouse model following therapeutic intra-nasal administration of IL-27. Based on the “Hygiene Hypothesis” in the early years, we propose that the presence of a Th1 environment before the asthma challenge may alleviate the onset of asthma in our model. To test this hypothesis, we administered IL-27 to OVA-immunized mice at two concentrations (25 and 50 ng/mouse) twice a day for 7 days before the OVA challenge (after the second OVA sensitization) (Additional file 1: Figure S1A), and there seemed to be no obvious improvement in the mouse asthma model (Additional file 1: Figure S1B, C). Then, we changed the intervention timing of the IL-27 administration. We administered IL-27 to OVA-immunized mice at the same concentration twice a day for 14 days before the second OVA sensitization (Fig. 3a). Notably, preventative i.n. administration of IL-27 before the second OVA sensitization dramatically attenuated airway inflammation, as evidenced by significantly decreased total cell numbers and differential cell counts, especially the eosinophils in BALF (Fig. 3b), as well as reduced peribronchial and perivascular inflammatory infiltrates observed in H&E-stained lung sections (Fig. 3e). Strikingly decreased accumulation of PAS-positive goblet cells was also observed in the low dose IL-27 treatment group (Fig. 3d). Consistently, there were diminished BALF concentrations of Th2 cytokines, such as IL-5 and IL-13, in the IL-27 preventative group (Fig. 3c). More importantly, preventative administration of IL-27 caused significant protection from methacholine-induced AHR, a hallmark of asthma (Fig. 3f). Together, these results demonstrate that preventative administration of exogenous IL-27 effectively alleviates Th2-mediated allergic asthma.

### Therapeutic administration of IL-27 neither reversed the impairment of the STAT1 pathway nor strengthened the GADD45γ/p38 MAPK pathway

**Fig. 4:**
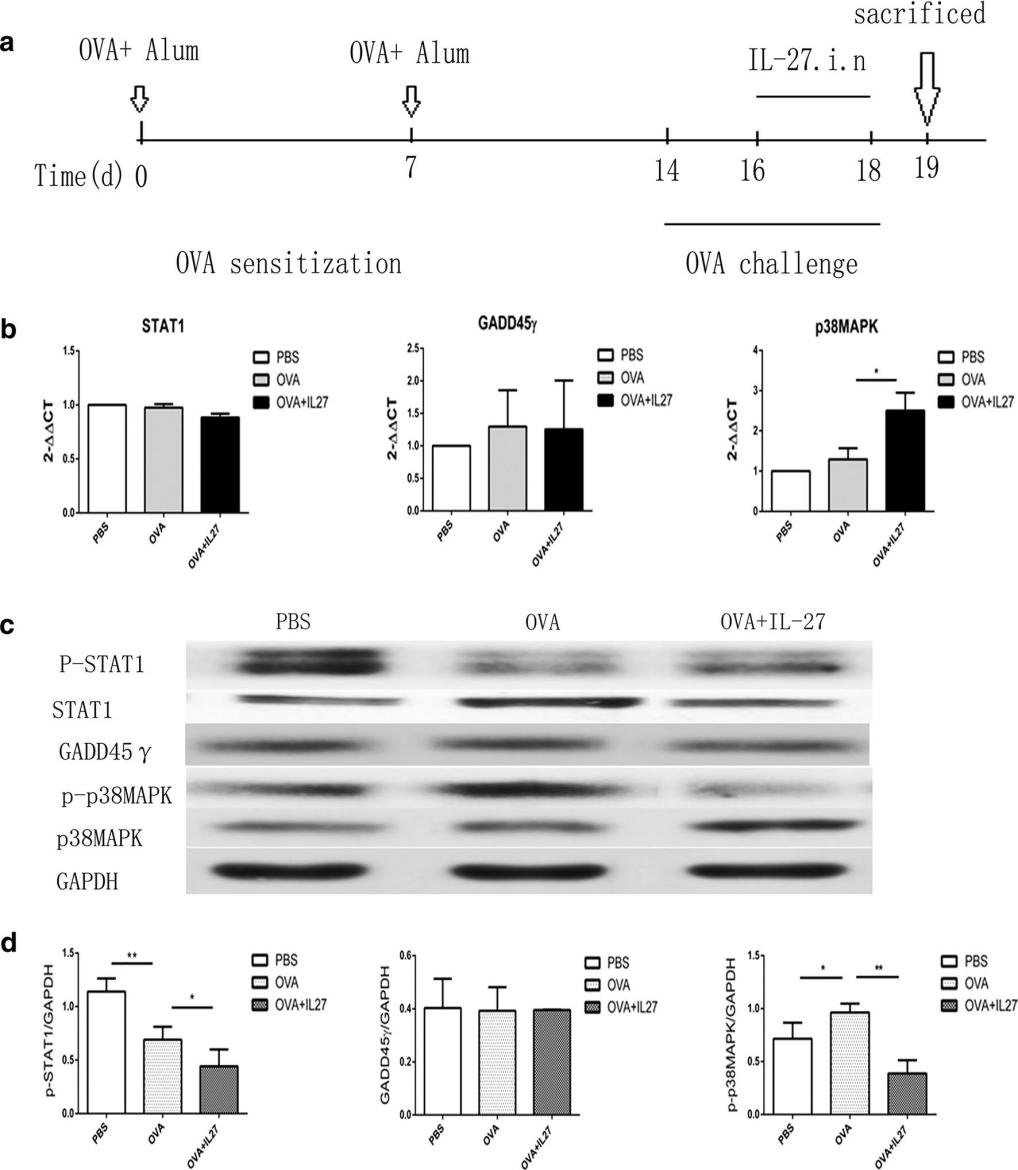
The STAT1 and GADD45γ/p38 MAPK pathways remain impairment in the IL-27 treatment groups. **a** Protocol of OVA-induced allergic asthma and IL-27 administration. **b** The mRNA expression of STAT1, GADD45-γ and p38 MAPK by qPCR. **c** The protein expression of STAT1, GADD45γ and p38 MAPK by Western blot. In IL-27 treated group, p-STAT1, p-p38MAPK were still impaired compared with PBS group, while GADD45γ had no markedly difference. **d** Densitometer measurements of pSTAT1/GAPDH, GADD45γ/GAPDH and p38 MAPK/GAPDH. IL-27 treatment had no improvement in the already impaired signal pathway.(GAPDH expression was acted as internal reference.) The *columns* and *error bars* represent the mean and SEM (n = 6 per group). *P < 0.05, **P < 0.01, ***P < 0.001. Similar results were obtained in at least three independent experiments

As demonstrated above, therapeutic administration of IL-27 in the challenge stage of asthma did not effectively suppresses Th2-mediated allergic asthma. Considering that IL-27 promotes the differentiation of Th1 while inhibiting the differentiation of Th2 via two pathways, STAT1 and GADD45γ/p38 MAPK [5, 11], we administered IL-27 to OVA-immunized mice in the last 3 consecutive days (Fig. 4a). We obtained the RNA and protein of lung tissues of each group and analyzed them using qPCR and Western blot. As shown, our data indicated that in the asthma group, the mRNA expression of STAT1, GADD45γand p38 MAPK exhibited no significant changes and that IL-27 intervention had no effect on STAT1 and GADD45γ but did up-regulate the expression of p38 MAPK (Fig. 4b). Consistently, the total protein expression of the three proteins exhibited corresponding changes (Fig. 4c, d). Simultaneously, the changes in the phosphorylation level of each protein were notable. In the asthma group, the phosphorylation level of STAT1 was significantly impaired, while p38 MAPK exhibited no obvious changes (there was a slight up-regulation). Interestingly, after the IL-27 treatment, the phosphorylation levels of the two proteins were significantly down-regulated. All of the results suggested that the inflammation existing in the asthma model mainly impaired the phosphorylation of the STAT1 protein, which resulted in the impairment of the STAT1 pathway, and had no obvious effect on the GADD45γ/p38 MAPK pathway. Therapeutic administration of IL-27 could neither reverse the impairment of the STAT1 pathway nor strengthen the GADD45γ/p38 MAPK pathway.

### Preventative administration of IL-27 strengthened both the STAT1 and GADD45γ/p38 MAPK pathways

**Fig. 5:**
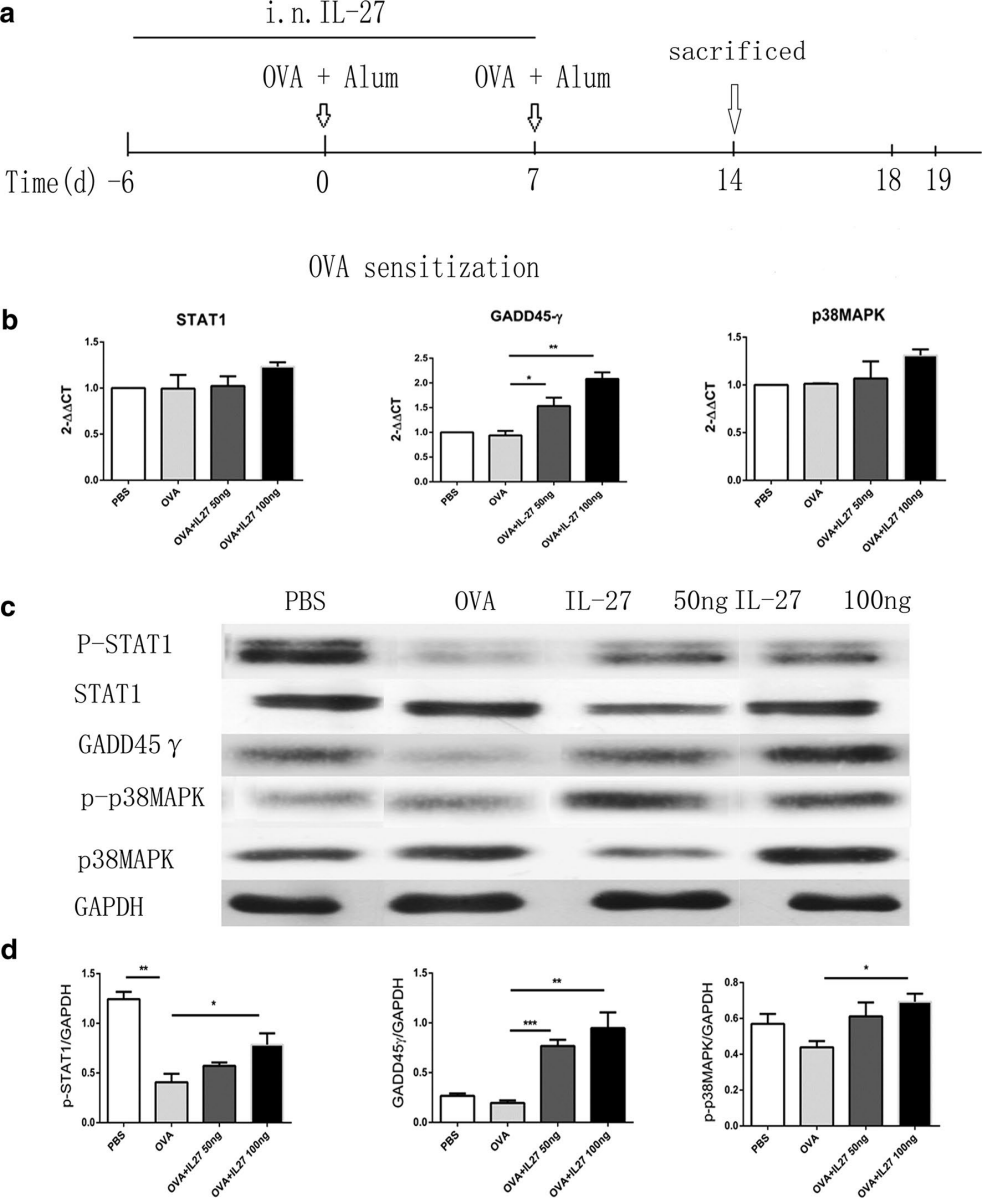
The STAT1 and GADD45γ/p38 MAPK pathways are up-regulated in the IL-27 prevention groups. **a** Protocol of OVA-induced allergic asthma and IL-27 administration. **b** The mRNA expression of STAT1, GADD45-γ and p38 MAPK by qPCR. It showed IL-27 preventative treatment reverse the impaired STAT1 and strengthen GADD45-γ expression as well. **c** The protein expression of STAT1, GADD45-γ and p38 MAPK by Western blot. It shows IL-27 preventative treatment reverse the impaired p-STAT1 and strengthen GADD45-γ/p38 MAPK protein expression as well. **d** Densitometer measurements of pSTAT1/GAPDH, GADD45-γ/GAPDH and p38 MAPK/GAPDH. GAPDH expression was acted as internal reference. The *columns* and *error bars* represent the mean and SEM (n = 6 per group). *P < 0.05, **P < 0.01, ***P < 0.001. Similar results were obtained in at least three independent experiments

In the last section, we verified that the impairment of the STAT1 and GADD45γ/p38 MAPK pathways had no effect on IL-27-mediated changes in the treatment group. Next, we further investigated whether the significant effect of IL-27 in the preventative group was related to the STAT1 and GADD45γ/p38 MAPK pathways. As per the protocol, we administered IL-27 i.n. to OVA-immunized mice before the OVA sensitization and sacrificed the mice before the OVA challenge (Fig. 5a). Next, we obtained the mRNA and protein of the lung tissues of each group for qPCR and Western blotting. The preventative administration of IL-27 obviously up-regulated the phosphorylation levels of the STAT1 and p38 MAPK proteins and the total protein of GADD45γin a dose-independent way, which was key for the activation of the STAT1 and GADD45γ/p38 MAPK pathways (Fig. 4c, d). In contrast, there was no significant up-regulation of the total protein and mRNA expression of STAT1 and p38 MAPK (Fig. 5b, c). These results suggest that preventative administration of IL-27 before OVA sensitization can either reverse the impairment of the STAT1 pathway or strengthen the GADD45γ/p38 MAPK pathway, presumably promoting Th1 differentiation and alleviating Th2-mediated allergic asthma.

## Discussion

IL-27, as a pleiotropic cytokine, works in promoting Th1 differentiation, whereas inhibiting Th2 differentiation [5]. Our study demonstrated IL-27 could suppress naïve CD4^+^ T cells different into Th2 cells, but not already committed Th2 cells’ redifferentiation in vivo [13]. We further administrated IL-27 intro-nasal to see whether IL-27 has the capacity to alleviate airway inflammation in asthma mice model. To our surprise, preventative administration of IL-27 via nose improved the pathological symptoms of OVA-induced asthmatic mice, but not the therapeutic treated group. STAT1 phosphorylation was impaired in the lung of mice in asthmatic condition. IL-27 was found to reverse STAT1 phosphorylation and strengthen GADD45/p38 MAPK pathway if it was given in a preventative way.

The inherent stability of Th2 poses a significant barrier to treating allergic diseases. Some research groups have previously studied the association between IL-27 and several Th2-mediated inflammatory diseases. L-27R–deficient mice displayed increased Th2 production when infected with the parasites of Leishmania major or *Trichuris muris* [6], and transgenic expression of IL-27 suppressed the Th2 responses induced by *Strongyloides venezuelensis* [7]. In a murine model of collagen-induced arthritis (CIA), short-term administration of IL-27 at the onset of the disease significantly attenuated the disease severity [21]. However, in another experimental arthritis model of proteoglycan-induced arthritis, it appears that IL-27 played a pro-inflammatory role [22].

Reports about the role of IL-27 in asthma are minimal. Yoshimoto and his team [7] reported that IL-27 suppressed Th2 cell development and Th2 cytokine production from polarized Th2 cells and that IL-27 could significantly improve the pathologic symptoms through large-dose i.n. administration during the OVA-challenge stage in an OVA-induced mouse asthma model. However, in our IL-27 treatment group, large-dose i.n. administration of IL-27 during the last 3 days of the OVA-challenge stage generated no significant improvement in the pathologic symptoms (Fig. 2). The differing results between Yoshimoto’s group and our study may be due to the difference between the protocols of our mouse asthma model (the former study used an asthma model of 10 days, and ours is 19 days), which may result in the different severity of Th2 inflammation.

In the present study, we proved that IL-27 did not inhibit the already differentiated Th2 cells in both mouse and human systems in vitro (Fig. 1) This might result in the inability of therapeutic i.n. administration of IL-27 to improve the pathological asthma symptoms. We propose that strengthening a Th1 environment before the asthma challenge model may alleviate the onset of asthma. To verify this hypothesis, we set up a low-dose preventative model with multiple administrations. In the beginning, we administered IL-27 for 7 days after OVA sensitization but before the OVA challenge and obtained no significant improvement in asthma symptoms (Additional file 1: Figure S1). We speculated that the failure effect may result from the inflammatory environment that had been built after the OVA sensitization. Thus, we changed the timing of the IL-27 administration and administered IL-27 for 14 days before the OVA sensitization (Fig. 3a). Notably, this protocol significantly improved the pathological change of asthma (Fig. 3).

STAT1 is particularly critically important for IL-27 signaling, leading to the up-regulation of ICAM-1 and T-bet expression in naive CD4^+^ T cells and, consequently, to Th1 differentiation [23]. Independently of STAT1, IL-27 also induced GADD45 expression, activating p38 MAPK [11], followed by augmentation of T-bet expression. Therefore, we further investigated whether the effect of IL-27 intervention was related to the two pathways by analyzing the mRNA and protein expression of STAT1, GADD45γ and p38 MAPK. We found that the inflammation existing in the asthma model mainly impaired the phosphorylation of the STAT1 protein, which resulted in the impairment of the STAT1 pathway, and had no obvious effect on the GADD45γ/p38 MAPK pathway. The large-dose therapeutic model of IL-27 with few administrations could neither reverse the impairment of the STAT1 pathway nor strengthen the GADD45γ/p38 MAPK pathway. In contrast, the low-dose preventative administration of IL-27 with multiple administrations could either reverse the impairment of the STAT1 pathway or strengthen the GADD45γ/p38 MAPK pathway and improve the pathologic symptoms of asthma. These results fully demonstrated the effect of IL-27 intervention through the STAT1 and GADD45γ/p38 MAPK pathways; Furthermore, these findings also suggest that IL-27 primarily promotes the differentiation of Th1 through STAT1 pathways in vivo.

Different methods of IL-27 administration resulted in two absolutely different effects. We propose that the key point is the timing of the administration of IL-27. In the treatment group, IL-27 was administered during the last 3 days of OVA challenge when the CD4^+^ T cells were already almost differentiated into Th2 cells, and the differentiated Th2 cells resisted the inhibitory effect of IL-27. In the prevention group, IL-27 was administered before OVA sensitization when the CD4^+^ T cells were largely naïve and could be promoted to differentiate into Th1 cells by IL-27, supporting an environment of Th1 cells before the asthma challenge could alleviate the onset of an asthma attack.

## Conclusions

In conclusion, preventative administration of IL-27 could either reverse the impairment of STAT1 or strengthen the expression of GADD45γ/p38 MAPK which leads to alleviate airway inflammation and improve AHR in asthmatic mice. Lack of effects in the therapeutic administration group contributed to already differentiated Th2 cells existing in asthmatic airways that resist to IL-27-mediated Th2 differentiation.

Our pilot study indicates a promising role for IL-27, a pleiotropic cytokine,in alleviating the pathogenesis of mouse asthma with the proper administration route and timing. This research sheds light on cytokine treatment, especially that involving IL-27, which has the potential to be used as an intervention in asthma prevention and management in the future.

## Additional file

10.1186/s12967-016-1039-x Preventative Intranasal administration of IL-27 after OVA sensitization had no improvement in an OVA-induced mouse model. (A) Protocol of OVA-induced allergic asthma and IL-27 administration. (B) The total cell number counts in bronchoalveolar lavage fluid (BALF). (C) Representative photomicrographs of lung sections stained with H&E. The Columns and error bars represent mean and SEM (n = 6 per group). *P < 0.05, **P < 0.01, ***P < 0.001. Similar results were obtained in at least six independent experiments. **Figure S2.** mRNA expression of GATA3 and T-bet of lung mononuclear cells by real-time PCR. A. GATA3 expression of lung mononuclear cells from PBS, OVA, OVA + IL-27 50 ng, OVA + IL-27 100 ng groups (HPRT mRNA level as an internal standard). B. T-bet expression of lung mononuclear cells from PBS, OVA, OVA + IL-27 50 ng, OVA + IL-27 100 ng groups (HPRT mRNA level as an internal standard). **P < 0.01 means significant difference.

## Abbreviations

IL-27: interleukin-27
OVA: ovalbumin
STAT: signal transducer and activator of transcription
GADD45γ: growth arrest and DNA damage 45-γ
p38 MAPK: p38 mitogen activated protein kinase
Th: T help
T-bet: T-box transcription factor
iCAM-1: intercellular cell adhesion molecule-1
LFA-1: lymphocyte function-associated antigen-1
IL-27R: IL-27 receptor
Al(OH)3(Alum): adjuvant aluminum hydroxide
rhIL-27: recombinant human IL-27 antibody
rmIL-27: recombinant mouse IL27 antibody
rhIL-4: recombinant human IL-4 antibody
rmIL-4: recombinant mouse IL-4 antibody
rhIL-2: recombinant human IL-2 antibody
rmIL-2: recombinant mouse IL-2 antibody
PBS: phosphate-buffered saline
i.n: intra-nasal
BAL: bronchoalveolar lavage
H&E: hematoxylin–eosin
PAS: periodic acid–Schiff
AHR: airway hyperreactivity
CIA: collagen-induced arthritis
