# Supplementary material for: IL-27 attenuates airway inflammation in a mouse asthma model via the STAT1 and GADD45γ/p38 MAPK pathways
Source: J Transl Med. 2016 Sep 29;14:283. doi: 10.1186/s12967-016-1039-x (PMC5041330; doi:10.1186/s12967-016-1039-x)
Supplement: Supplementary file 1 — 10.1186/s12967-016-1039-x Preventative Intranasal administration of IL-27 after OVA sensitization had no improvement in an OVA-induced mouse model. (A) Protocol of OVA-induced allergic asthma and IL-27 administration. (B) The total cell number counts in bronchoalveolar lavage fluid (BALF). (C) Representative photomicrographs of lung sections stained with H&E. The Columns and error bars represent mean and SEM (n = 6 per group). *P < 0.05, **P < 0.01, ***P < 0.001. Similar results were obtained in at least six independent experiments. Figure S2. mRNA expression of GATA3 and T-bet of lung mononuclear cells by real-time PCR. A. GATA3 expression of lung mononuclear cells from PBS, OVA, OVA + IL-27 50 ng, OVA + IL-27 100 ng groups (HPRT mRNA level as an internal standard). B. T-bet expression of lung mononuclear cells from PBS, OVA, OVA + IL-27 50 ng, OVA + IL-27 100 ng groups (HPRT mRNA level as an internal standard). **P < 0.01 means significant difference. [file 12967_2016_1039_MOESM1_ESM.docx]

Fig S1. Preventative Intranasal administration of IL-27 after OVA sensization had no improvement in an OVA-induced mouse model. (A) Protocol of OVA-induced allergic asthma and IL-27 administration. (B) The total cell number counts in bronchoalveolar lavage fluid (BALF). (C)Representative photomicrographs of lung sections stained with H&E. The Columns and error bars represent mean & SEM (n = 6 per group). *P < 0.05, **P < 0.01, ***P < 0.001. Similar results were obtained in at least six independent experiments.


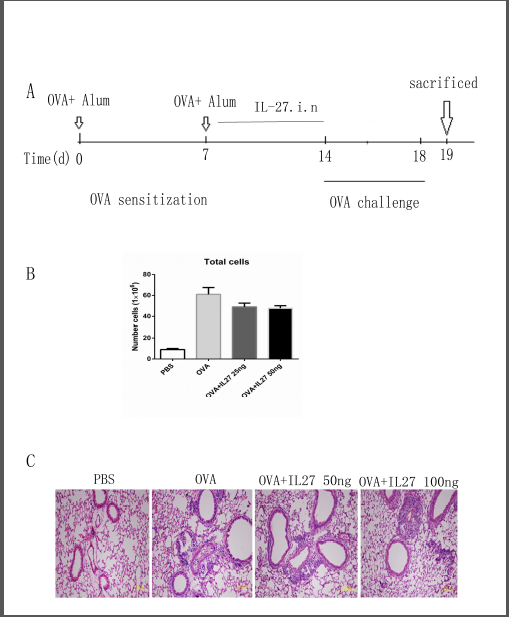


Fig S2. mRNA expression of GATA3 and T-bet of lung mononuclear cells by real-time PCR. A. GATA3 expression of lung mononuclear cells from PBS, OVA, OVA+IL-27 50ng, OVA+IL-27 100ng groups (HPRT mRNA level as an internal standard). B. T-bet expression of lung mononuclear cells from PBS, OVA, OVA+IL-27 50ng, OVA+IL-27 100ng groups (HPRT mRNA level as an internal standard). **P < 0.01 means significant difference.
